# Quantitative genetics of breeding coloration in sand lizards; genic capture unlikely to maintain additive genetic variance

**DOI:** 10.1038/s41437-023-00607-8

**Published:** 2023-03-20

**Authors:** Willow R. Lindsay, Badreddine Bererhi, Gabriella Ljungström, Erik Wapstra, Mats Olsson

**Affiliations:** 1 Department of Biological and Environmental Sciences, Göteborg University, Göteborg, Sweden; 2 Department of Biological Sciences, University of Bergen, Bergen, Norway; 3 School of Natural Sciences, University of Tasmania, Hobart, TAS Australia

**Keywords:** Sexual selection, Evolutionary genetics, Quantitative trait

## Abstract

Sexual selection on fitness-determining traits should theoretically erode genetic variance and lead to low heritability. However, many sexually selected traits maintain significant phenotypic and additive genetic variance, with explanations for this “lek paradox” including genic capture due to condition-dependence, and breaks on directional selection due to environmental sources of variance including maternal effects. Here we investigate genetic and environmental sources of variance in the intrasexually selected green badge of the sand lizard (*Lacerta agilis*). The badge functions as a cue to male fighting ability in this species, and male–male interactions determine mate acquisition. Using animal models on a pedigree including three generations of males measured over an extensive 9-year field study, we partition phenotypic variance in both badge size and body condition into additive genetic, maternal, and permanent environmental effects experienced by an individual over its lifespan. Heritability of badge size was 0.33 with a significant estimate of underlying additive genetic variance. Body condition was strongly environmentally determined in this species and did not show either significant additive genetic variance or heritability. Neither badge size nor body condition was responsive to maternal effects. We propose that the lack of additive genetic variance and heritability of body condition makes it unlikely that genic capture mechanisms maintain additive genetic variance for badge size. That said, genic capture was originally proposed for male traits under female choice, not agonistic selection. If developmental pathways generating variance in body condition, and/or the covarying secondary sex trait, differ between inter- and intrasexual selection, or the rate at which their additive genetic variance or covariance is depleted, future work may show whether genic capture is largely restricted to intersexual selection processes.

## Introduction

Strong directional selection on sexually selected signals should erode trait variance and result in a loss of underlying additive genetic variance and low heritability (Van Homrigh et al. 2007). In contrast to predictions, variance in sexually selected traits and underlying genetic variance often remains remarkably high (Pomiankowski and Møller 1995). Several hypotheses have been proposed to resolve this “lek paradox” (Kirkpatrick and Ryan 1991; Pomiankowski and Møller 1995; Rowe and Houle 1996), yet empirical support from wild populations lags far behind theoretical explanations, and the relative importance of environmental vs. genetic effects in determining sexually selected trait variance remains unclear (Howie et al. 2019; Martinossi-Allibert et al. 2018; Qvarnström and Price 2001).

The genic capture hypothesis offers a resolution to the lek paradox, proposing that when sexually selected traits are condition-dependent and polygenic aspects of underlying condition are themselves heritable, significant additive genetic variance can be maintained through a mutation-selection balance (Kotiaho et al. 2001; Rowe and Houle 1996; Tomkins et al. 2004). Genomic evidence from 14 generations of *Drosophila melanogaster*, for example, support a genic capture resolution to the lek paradox, showing both erosion of genetic variance arising from sexual selection and a molecular signature of genic capture, likely mediated through selection on body condition which acts as a constraint on loss of genetic variance (Dugand et al. 2019). How taxonomically widespread such phenomena might remain unclear (Baur and Berger 2020; Schielzeth et al. 2012). In addition, since sexually selected traits are often controlled by genes at multiple loci, a significant additive genetic variance may persist (Merila and Sheldon 1999) without necessitating a condition-dependent link (e.g., Schielzeth et al. 2012).

How a sexually selected trait responds to selection hinges not only on heritability, the proportion of the phenotypic variance in a trait that is due to additive genetic effects (Falconer and Mackay 1996), but also on nongenetic environmental and maternal effects (Griffith et al. 1999; Qvarnström and Price 2001). Females influence the phenotype of their offspring through the developmental environment or differential allocation of maternal resources (e.g., hormones, antioxidants, fatty acids; Mentesana et al. 2019; Mousseau and Fox 1998). These effects may counteract the depletion of additive genetic variance arising from the directional selection on sexually selected traits (Bonduriansky and Day 2009). In house sparrows (*Passer domesticus*), for example, sexually selected badge size of cross-fostered offspring more closely resembles the badge size of their foster fathers than biological fathers, a result that demonstrates the strong contribution of environmental and parental rearing conditions on sexually selected trait development (Griffith et al. 1999). Yet house sparrow badge size itself has a significant additive genetic component and a low but significant estimate of heritability (Jensen et al. 2008).

We use quantitative genetic “animal models” (Lynch and Walsh, 1998; Kruuk 2004) to examine genetic, maternal, and phenotypic components of variance underlying expression of the sexually selected green ventral badge of the Swedish sand lizard (*Lacerta agilis*). Badge size is an intrasexually selected signal of male fighting ability in this species (Olsson 1994a, b) that is strongly linked to fitness (Anderholm et al. 2004; Olsson 1994a; Olsson et al. 2000, 2005). Selection gradient analyses reveal significant directional selection on badge size (Olsson 1994a), predictive of erosion in underlying additive genetic variance. Directional selection on badge size drives mate acquisition (Anderholm et al. 2004), but other mechanisms may drive mating and/or fertilization success (Olsson et al. 2005). Since badge size is associated with body condition (Anderholm et al. 2004; Lindsay et al. 2016; Olsson 1994a), the expression of this ornament may genetically “capture” aspects of male condition in this species (as per Rowe and Houle 1996), resulting in maintenance of genetic variance. Because badge size predicts male performance, with larger-badged males more likely to win aggressive interactions (Olsson et al. 2005), it is also likely that variance in badge size responds to selection pressures important for the maintenance of other endurance-related traits. Maternal effects are known to influence phenotypic traits related to body size and physical performance in lizards (Shine and Harlow 1993, Paranjpe et al. 2013, Noble et al. 2014). In the Eastern water skink (*Eulamprus quoyii*), for example, fitness-determining motor behaviors of offspring are more strongly influenced by maternal effects than additive genetic variance (Noble et al. 2014). We therefore include maternal identity in our models when appropriate in order to avoid artificial inflation of estimates of additive genetic variance (Kruuk and Hadfield 2007), which may occur when maternal effects are significant and not accounted for. We use 9 years of badge size and body condition data from a long-term study population of Swedish sand lizards. These morphological measurements are paired with high-resolution genetic paternity (Olsson et al. 2011), enabling us to assess the validity of the genic capture hypothesis by (i) estimating additive genetic variance and heritabilities for badge size and body condition, (ii) partition genetic vs. environmental sources of variance in badge size and body condition, and (iii) by attempting to quantify a genetic correlation between badge size and body condition.

## Methods

### Study species, field methods and animal husbandry

Sand lizards are small (max weight ca. 20 g), sexually dimorphic ground-dwelling lizards distributed primarily across central Europe but also extending into the UK, Sweden and central Asia (Aghasyan et al. 2021). Here we present data on male badge size and body condition from male sand lizards captured between 2000 and 2008 from our long-term field site located at Asketunnan on the Swedish west coast.

Male sand lizards develop a green ventro-lateral badge of color following emergence from hibernation while females in our study population maintain the brown and cream non-breeding coloration shared between the sexes.

**Fig. 1:**
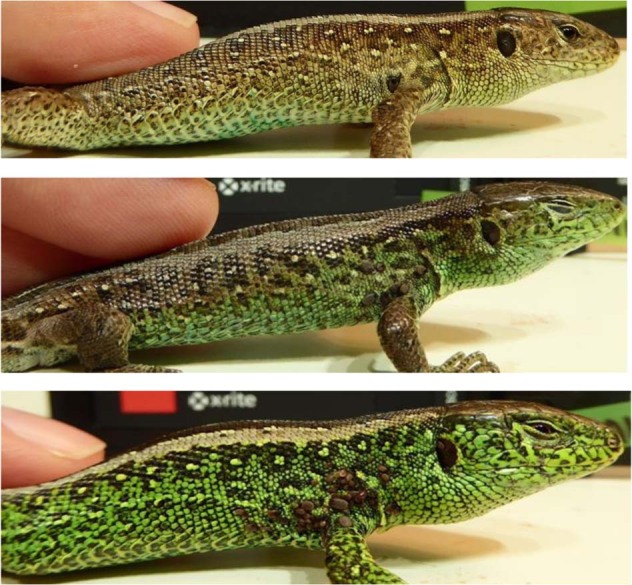
Males with different sized badges from small to large (top to bottom). The badges were measured as proportions of green color from a flatbed scanned image.

**Fig. 2:**
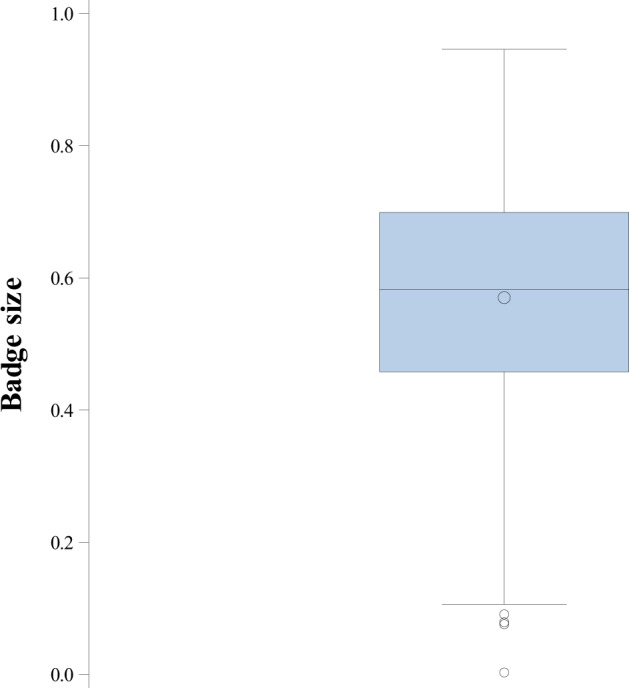
Estimates of badge size in the study population. Descriptive box plot of badge size estimates (mean proportion of badge size, ±SE, *N* = 217).

Our field protocols and animal husbandry techniques have been published elsewhere (Olsson 1994a; Lindsay et al. 2020; Olsson et al. 2000), and thus we provide only brief detail here. We captured males and females by noose, collected blood from the *sinus angularis*, and measured snout-vent length (mm; SVL) and body mass to the nearest 0.001 g. We measured badge size by scanning each male on a flatbed scanner, and calculating the proportion (%) of green coloration versus the remaining side of the body (Olsson and Madsen 2001) (Figs. 1 and 2). We brought females into captivity when visibly gravid (egg contours apparent along their abdomens), and kept them in individual terraria (10 × 50 × 60 cm) until oviposition. Each cage was fitted with a flat rock, wet sand for egg laying, and a 40 W spotlight to allow for thermoregulation. We collected eggs following lay and incubated them at their temperature optimum of 25 °C (Zakharov 1989). Females were immediately released back to their capture locations. After the egg hatched (ca. 40 days), we collected offspring tail tips (that re-grow) for genetic paternity analyses and released young at random locations on the Asketunnan study site. Constant incubation temperature across years, an approximate 2-year lag phase between incubation year and maturation (badge development), and a random release in relation to habitat quality (and most likely its influence on body condition) speak strongly against artificial incubation effects on the environmental and genetic effects we quantify on badge size, body condition and their interactions.

### Genetic paternity analysis

We determined parentage using 17–21 microsatellite loci following detailed methods that are published elsewhere (Olsson et al. 2011; for summary, see electronic supplement). Our final pedigree presented in this study contains 138 adult males with known parentage over three generations, incorporating 87 fathers and 84 mothers.

### Statistical analyses

**Fig. 3:**
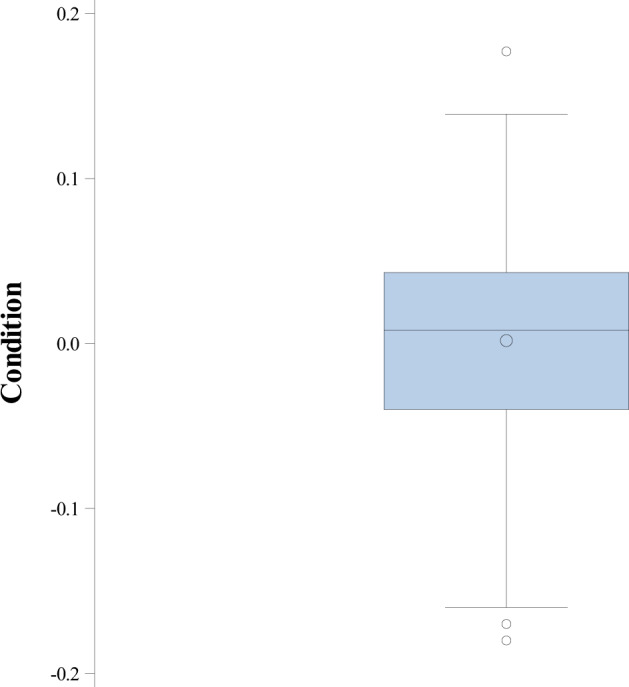
Estimates of body condition in the study population. Descriptive box plot of body condition (residuals from mass-snout-vent length regression, *N* = 231).

We estimated the condition as the residuals of a regression of the cube root of body mass on SVL (Fig. 3). We include SVL in badge size models, given the significant associations between these variables (Olsson and Shine 1996). Given the variance in badge size based on the date of capture, we add the date of capture as a covariate in all badge size analyses.

We examined phenotypic variance (*V*_*P*_) and calculated estimates of heritability (*h*^2^) for badge size and body condition using animal models (Lynch and Walsh 1998; Kruuk 2004). These analyses, which incorporate pedigree information, allow partitioning of variance in traits between additive genetic effects (*V*_*A*_), nongenetic effects due to maternal identity (*V*_*MA*_), and effects due to permanent environment experienced over a lifespan by an individual (*V*_*PE*_). Our models represent the total phenotypic variance as *V*_*P*_ = *V*_*A*_ + *V*_*MA*_ + *V*_*PE*_ + *V*_*R*_, where *V*_*R*_ encompasses residual variance. In each model, we also include fixed effects and covariates known to influence trait variance (e.g., year, date of capture, body condition, and SVL). We tested for the significance of random effects components (*V*_*A*_, *V*_*PE*_, *V*_*MA*_) using log-likelihood ratio test comparisons of models with and without each random effect, and a significance threshold of *p* < 0.05. When the random effect for *V*_*MA*_ was not significant following log-likelihood ratio testing, it was excluded from the final model (as per Martins et al. 2019). We calculate heritability as $h^2 = {\textstyle{{V_A} \over {V_P}}}$. All statistical tests were performed in ASReml-W release 4.1.

## Results

We found a significant additive genetic variance component to badge size with a heritability estimate of 0.33 (Table 1). Other fixed effects and covariates, including year, date of capture, and SVL, significantly influenced badge size, but body condition was not a significant predictor in this model (*p* = 0.252). Maternal ID (*V*_*MA*_) was non-significant (estimate = 0.0036 ± 0.002 SE, *n* = 84, *p* = 0.125) and was dropped from the final model.

**Table 1:** Heritability estimates and phenotypic variance components for badge size and body condition.

Response	Parameter	Estimate	SE	*n*	*χ*^*2*^ (*p*)
Badge size (*n* = 212 observations of 138 males)					
	*V*_*R*_	0.009	0.001	212	–
	*V*_*P*_	0.018	0.002	–	–
	*V*_*PE*_	0.0003	0.003	138	0.006 (0.47)
	*V*_*MA*_	≈0	–	83	0 (0.50)
	*V*_*A*_	0.006	0.004	138	3.30 **(0.03)**
	*h*^2^	0.33	0.20	–	–
	*V*_Year_	0.002	0.001	9	20,176 **(<0.001)**
	Date	*F*_32, 143.9_ = 3.67	–	35	**<0.001**
	Snout-vent length	0.090	0.010	212	**<0.001**
	Condition	0.015	0.011	212	0.16
Body condition (*n* = 231 observations of 138 males)					
	*V*_*R*_	0.0014	0.0002	231	–
	*V*_*P*_	0.0044	0.0007	–	
	*V*_*PE*_	0.0020	0.0008	138	6.99 **(0.004)**
	*V*_*MA*_	≈0	–	83	0 (0.50)
	*V*_*A*_	0.00002	0.0007	138	0.002 (0.48)
	*h*^2^	0.0053	0.1696	–	–
	*V*_Year_	0.0009	0.0005	9	28.79 **(<0.001)**
	Date	*F*_31,135.8_ = 1.94	–	35	**0.005**

Output from two animal models is provided, including model estimates, standard errors, factor sample sizes, and *p* values with significant terms bolded. Fixed effects and covariates are listed below random effects components and estimates of heritability. Term abbreviations are as follows: residual variance (*V*_*R*_), phenotypic variance (*V*_*P*_), permanent environment effects (*V*_*PE*_), maternal effects (*V*_*MA*_) additive genetic effects (*V*_*A*_), year effects (*V*_Year_), narrow sense heritability (*h*^2^), standard error (SE), sample size (*n*), *p* value (*p*). Phenotypic variance was estimated as *V*_*P*_ = *V*_*R*_ + *V*_*A*_ + *V*_*MA*_ + *V*_*PE*_ + *V*_*R*_ + *V*_Year_. Narrow sense heritability was calculated using the formula ${{h}}^2 = {\textstyle{{{{V_A}}} \over {{{V_P}}}}}$. For variance parameters, the *χ*^2^ statistics, obtained from log-likelihood ratio tests, are added followed by their corresponding *p* values in parentheses. All log-likelihood ratio tests were performed using one degree of freedom (the difference in the number of variance components between models). Note that the *p* values were divided by 2 for the hypothesis testing of variance parameters.

Variance in body condition was significantly affected by the permanent environment and the fixed effects of year and capture date (Table 1). We found a low and non-significant signature of additive genetic variance (0.00002), and a low heritability (0.005) of body condition (Table 1). Maternal identity (*V*_*MA*_) was non-significant (estimate = 0, *p* = 0.5) and excluded from the final model, although its inclusion did not alter any other variance estimates.

During these analyses, we also made attempts at quantifying the genetic correlation between body condition and badge size, but unfortunately, these models did not converge and will not be further reported on.

## Discussion

We found significant additive genetic variance underlying the sexually selected sand lizard badge. Genic capture is an unlikely resolution to this maintenance of genetic variance in the face of selection (i.e., lek paradox), since body conditions show low and non-significant additive genetic variance, with variance significantly predicted by the environmental conditions experienced over an individual’s lifetime. We propose that complex genetic, structural and pigmentary mechanisms involved in the production of the multicomponent badge, along with age-dependent and sex-specific variance in selection on badge size, act as a check on loss of genetic variance for this fitness-determining trait. Moreover, intrasexual selection on the male badge, which drives access to mates (Anderholm et al. 2004; Olsson 1994a), may be diluted by pre- or post-copulatory mate-choice selection, e.g., on MHC dissimilarity (Olsson et al. 2005), overall reducing the strength of selection on badge size and offsetting loss to underlying genetic variance.

Both the strength and direction of selection on heritable traits can vary within an individual’s lifespan (e.g., Bourret et al. 2017) and offset erosion on additive genetic variance. In sand lizards, it was only the oldest males (age 7+) for which selection gradient analysis revealed significant selection on badge size (Olsson 1994a). In contrast to these larger, older males, young males face trade-offs between somatic growth and energetic investments in reproductive behavior and pigmentation (Olsson 1994b). Notably, however, these selection analyses were performed on mate acquisition only, not molecular paternity data (currently in progress). Artificial enlargement of young but not old male badges significantly increases mating success and body condition, effectively allowing these males to sidestep costly investments into ornament production (Anderholm et al. 2004). These trade-offs may undermine the strength of selection on badge size over a lifetime and contribute to the maintenance of phenotypic (and genetic) variance in this fitness-determining trait. For example, in the common lizard (*Lacerta vivipara*), the strength of selection on fitness-linked physical endurance capacity varies across age classes, with positive viability selection apparent in juveniles and yearlings only and weak sexual selection in adult males. These relationships were non-linear, and likely permissive to the high heritability of locomotor performance in this species (Le Galliard and Ferrière 2008).

In this study, maternal effects on phenotypic variance in badge size (as well as body condition) were negligible and therefore unlikely to influence our estimate of badge size heritability (Kruuk and Hadfield 2007). However, we contend that maternal genes and selection of females may be important contributors to genetic variance underlying badge size. Green coloration, while largely sex-limited with exaggeration highest in males, is a shared trait in this species (expressed also in females at artificial outbreeding; Lindsay et al. pers. obs.). Shared traits show more additive genetic variance than sex-specific traits, likely as a result of differences in the strength of selection between the sexes with weaker selection in females offsetting erosion of genetic variance and reduction in heritability estimates (Cally et al. 2019; Singh and Punzalan 2018). Moreover, the ZW/ZZ female heterogametic sex chromosome system exhibited by sand lizards is associated with genetic architecture that shows greater heritability of fitness-determining traits than for male heterogametic sex chromosome systems (Connallon 2010), with the allelic diversity underlying male ornamentation more resilient to loss in these systems (Reeve and Pfennig 2003).

Unlike badge size, male body condition has a very little additive genetic variance, instead reflecting nongenetic factors related to variance in the environmental conditions experienced over an individual’s lifespan. Such environmental effects might include variance in climatic conditions, food abundance and nutritional state, or parasite load. For example, sand lizard body condition varies with both exposure to ectoparasites (ticks) and physiological response to parasite load through the production of corticosteroid stress hormones (Lindsay et al. 2016). Male badge size has direct effects on body condition, with experimental badge enlargement increasing body condition, likely as a consequence of better access to food resources (Anderholm et al. 2004). Thus, directionality in the relationship between badge size and our measure of body condition may explain the lack of condition-dependence documented here despite previous research showing the reverse (Anderholm et al. 2004; Lindsay et al. 2016; Olsson 1994a). Furthermore, in previous work using a larger sample (with no constraint from genetic information), we found a significant effect of body condition on badge size (Olsson 1994a). Thus, it appears that the lack of significant effect of condition on badge size reported here may be due to the relatively small sample of adult males that was available for the pedigree.

The condition can be measured in a variety of ways, and while some metrics of condition may be more responsive to environmental conditions, others may be more affected by genetic traits (e.g., Merilä et al. 2001). For example, in Alston’s singing mice (*Scotinomys teguina*), condition metrics that reflect body composition showed higher estimates of heritability than those that described variance in metabolic responses to feeding vs. fasting. It was for these body composition metrics of condition that genetic correlations with sexually selected singing behavior indicated that heritability in song rate might be “captured” by condition (Burkhard et al. 2020). Further tests of the genic capture hypothesis in the sand lizard would benefit from the collection of other physiological metrics of the condition, such as concentrations of plasma lipids necessary for storage in adipose tissue, along with genetic and phenotypic correlations between their expression and the expression of the sexually selected badge.

Differences in estimates of heritability may exist between the size and pattern of a color badge and the reflectance parameters of that patch (Karino and Haijima 2001). Estimates of the heritability of various color patches on a single individual may also differ based on the signaling content of each patch (Tibbetts 2010). Elaborate coloration is comprised of multiple elements including various pigments (e.g., melanins and carotenoids), structural components, and patterns that can signal different aspects of individual quality and as such, predictions concerning their underlying genetic architecture and heritability may vary. Since badge size itself reflects varying investments in both structural and pigmentary components, our estimate of badge size heritability and genetic variance would also include structural and pigmentary contributions. While we show no influence of genic capture on the maintenance of additive genetic variance for overall badge size in sand lizards, sources of variance in the color or “greenness” of the badge remain to be explored. The multicomponent nature of the badge itself may speak to the presence of complex genetic pathways involved in badge development important for the maintenance of additive genetic variance in this trait. Future research would benefit from targeting the genetic and genomic interactions, and “genic capture” constraints, regulating the developmental pathways of the trait(s) themselves under directional selection, rather than “body condition” per se. Finally, if the rate at which the additive genetic variance or covariance between an ornament and body condition (or its components) differ in rate of depletion between intra- and intersexually selected traits, we may find different patterns in how genic capture plays a role in explaining the maintenance of genetic variance in secondary sex traits. A way forward in future work is to partition out loss of genetic variance in relation to, preferably experimentally manipulated, male–male competition and female choice scenarios and their interactions (for a lucid description of approaches, see Hunt et al. 2008). This may reveal that genic capture processes serve a more major role in maintaining genetic variance in male secondary sex traits in systems with strong, “unfiltered” directional selection, such as in female choice on immediate “run-away” traits in lekking species, rather than in traits involved in agonistic male–male competition.

## Supplementary information

Electronic supplement

## Data Availability

The data used in this study are available on Dryad at 10.5061/dryad.gf1vhhmtj.
