# Supplementary figures and images for: Quantitative genetics of breeding coloration in sand lizards; genic capture unlikely to maintain additive genetic variance
Source: Heredity (Edinb). 2023 Mar 20;130(5):329–34. doi: 10.1038/s41437-023-00607-8 (PMC10162981; doi:10.1038/s41437-023-00607-8)

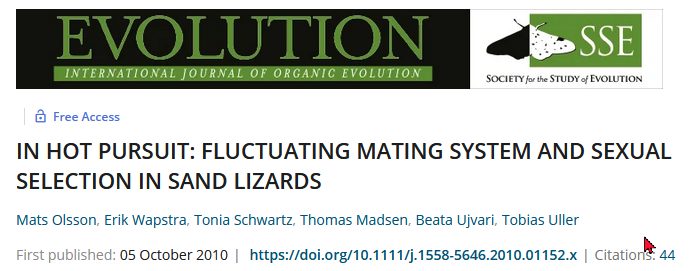


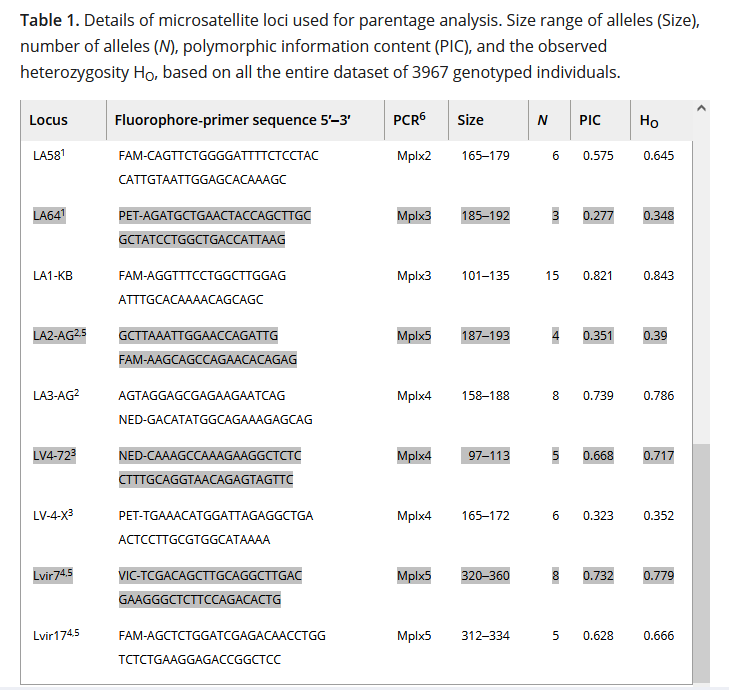

Supplement: Supplementary file 1 — Electronic supplement [file 41437_2023_607_MOESM1_ESM.docx]
